# P-1156. In Vitro Antimicrobial Activity of Cefepime in Combination with Taniborbactam Against Resistant Clinical Gram-negative Isolates from a Global Collection, 2018-2023

**DOI:** 10.1093/ofid/ofaf695.1349

**Published:** 2026-01-11

**Authors:** Mark G Wise, Meredith Hackel, Daniel F Sahm

**Affiliations:** IHMA, Schaumburg, IL; IHMA, Schaumburg, IL; IHMA, Schaumburg, IL

## Abstract

**Background:**

Taniborbactam is a novel broad-spectrum β-lactamase inhibitor with inhibitory activity against both serine- and metallo-β-lactamases. Taniborbactam restores the activity of cefepime (FEP) against many difficult to treat organisms, including cephalosporin- and carbapenem-resistant Enterobacterales (EB) and *Pseudomonas aeruginosa* (PA). The activities of cefepime-taniborbactam (FTB) and comparators were evaluated against nonsusceptible (NS)/resistant (R) clinical isolates of EB and PA collected for the Global Evaluation of Antimicrobial Resistance via Surveillance (GEARS) program.

**Figure:**
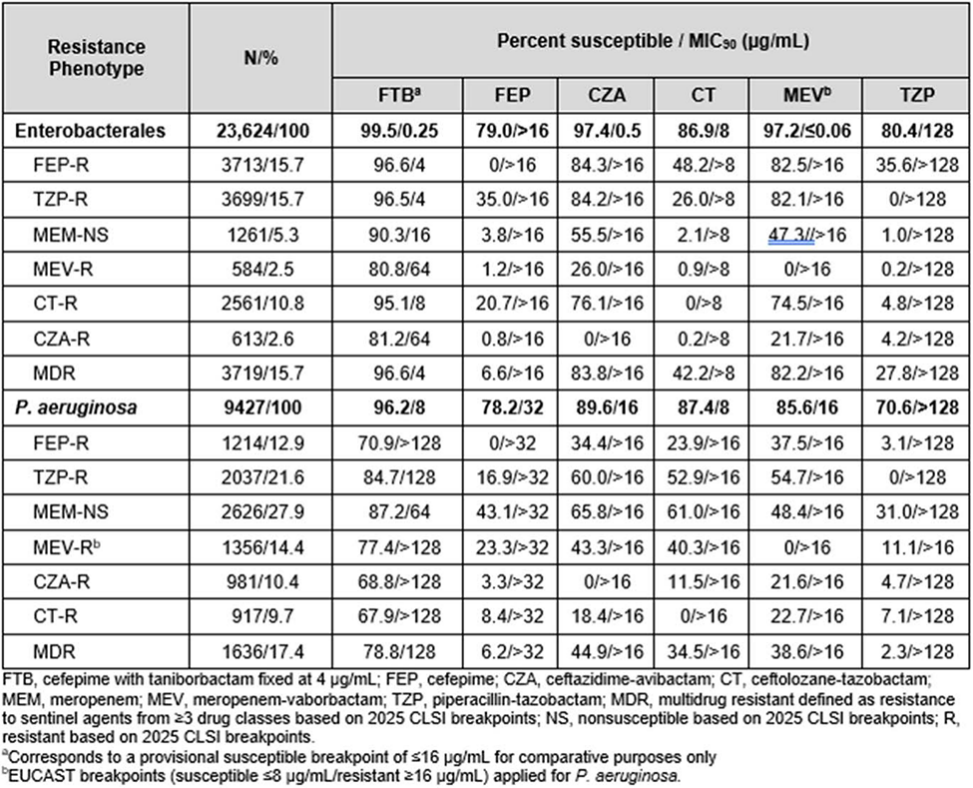

**Methods:**

MICs of FTB (with taniborbactam fixed at 4 µg/mL) and comparators were determined by broth microdilution (CLSI M07Ed12) against EB (n=23,624) and PA (n=9,427) collected from 351 clinical sites in 62 countries from 2018-2023. For FTB, a provisional susceptible breakpoint of ≤16 µg/mL was used for comparative purposes. NS/R phenotypes were based on 2025 CLSI breakpoints (EUCAST breakpoint for meropenem-vaborbactam [MEV] against PA). Multidrug R (MDR) was defined as R to sentinel agents from ≥3 drug classes.

**Results:**

Similar percentages (15.7%) of EB isolates were R to FEP and piperacillin-tazobactam (TZP), (Table). FTB had potent activity against all EB (MIC_90_, 0.25 µg/mL; 99.5% inhibited at ≤16 µg/mL). FTB maintained activity against >90% of meropenem (MEM)-NS, and ceftolozane-tazobactam (CT)-R EB, and >80% of MEV- and ceftazidime-avibactam (CZA)-R isolates. FTB at ≤16 µg/mL inhibited 96.6% of EB identified as MDR. FTB was the most active agent against PA overall (MIC_90_, 8 µg/mL; 96.2% inhibited at ≤16 µg/mL). Among MEM-NS PA isolates, 87.2% were inhibited by FTB at ≤16 µg/mL compared to 61.0% susceptible to CT. FTB at ≤16 µg/mL inhibited 67.9% of CT-R isolates whereas 18.4% and 22.7% of these isolates were susceptible to CZA and MEV, respectively. Against MDR PA (17.4% of all PA), FTB inhibited 78.8% at ≤16 µg/mL compared to 34.5% susceptible to CT.

**Conclusion:**

FTB had potent *in vitro* activity against worldwide EB and PA, including MDR isolates and isolates R/NS to FEP, TZP, MEM, MEV, CT, and/or CZA. These data support continued development of FTB as a potential treatment option for challenging infections due to resistant Gram-negative pathogens.

**Disclosures:**

Mark G Wise, PhD, IHMA: Employee

